# The Biological and Ecological Features of Northbound Migratory Birds, Ticks, and Tick-Borne Microorganisms in the African–Western Palearctic

**DOI:** 10.3390/microorganisms11010158

**Published:** 2023-01-07

**Authors:** Tove Hoffman, Björn Olsen, Åke Lundkvist

**Affiliations:** 1 Zoonosis Science Center, Department of Medical Biochemistry and Microbiology, Uppsala University, 751 23 Uppsala, Sweden; 2 Zoonosis Science Center, Department of Medical Sciences, Uppsala University, 751 85 Uppsala, Sweden

**Keywords:** African–Western Palearctic, migratory birds, *Hyalomma*, *Ixodes*, Alkhurma (Alkhumra) hemorrhagic fever virus, Crimean–Congo hemorrhagic fever virus, *Anaplasma phagocytophilum*, spotted fever group *Rickettsia*, *Francisella tularensis*, *Francisella*-like endosymbionts, *Midichloria*

## Abstract

Identifying the species that act as hosts, vectors, and vehicles of vector-borne pathogens is vital for revealing the transmission cycles, dispersal mechanisms, and establishment of vector-borne pathogens in nature. Ticks are common vectors for pathogens causing human and animal diseases, and they transmit a greater variety of pathogenic agents than any other arthropod vector group. Ticks depend on the movements by their vertebrate hosts for their dispersal, and tick species with long feeding periods are more likely to be transported over long distances. Wild birds are commonly parasitized by ticks, and their migration patterns enable the long-distance range expansion of ticks. The African–Palearctic migration system is one of the world’s largest migrations systems. African–Western Palearctic birds create natural links between the African, European, and Asian continents when they migrate biannually between breeding grounds in the Palearctic and wintering grounds in Africa and thereby connect different biomes. Climate is an important geographical determinant of ticks, and with global warming, the distribution range and abundance of ticks in the Western Palearctic may increase. The introduction of exotic ticks and their microorganisms into the Western Palearctic via avian vehicles might therefore pose a greater risk for the public and animal health in the future.

## 1. Introduction

The aim of this review is to increase the knowledge about the biological and ecological features of migratory birds, Ixodidae ticks, and tick-borne microorganisms—especially concerning birds in the African–Western Palearctic and the ticks and tick-borne microorganisms they transport during their northbound spring migration.

### 1.1. Zoonotic Diseases

Infectious diseases are a burden for both human and animal health globally. Among emerging infectious diseases, approximately 75% are zoonotic (i.e., transmitted between vertebrate animals and humans), and all originate in wildlife [1]. The World Health Organization (WHO), the Food and Agriculture Organization of the United Nations (FAO), and the World Organization for Animal Health (OIE) have defined an emerging zoonosis as “a zoonosis that is newly recognized or newly evolved, or that has occurred previously but shows an increase in incidence or expansion in geographical, host or vector range” [1]. Zoonotic infectious diseases have multiple routes of transmission, and a zoonotic pathogen can be transmitted to humans directly from a vertebrate host, indirectly via contaminated food or water, or via a vector. The rapid expansion of the human population has led to urbanization, and the emergence of many zoonotic diseases can be linked to anthropogenic factors (caused by humans or human activity), such as animal husbandry, habitat change, global travel, and trade. These factors have increased the interface between wildlife, domestic animals, and humans and provided more opportunities for spill-over events.

### 1.2. Vector-Borne Infections

A vector is an invertebrate that acts as a transmitter of an infectious agent between vertebrate hosts (organisms supporting replication of an infectious agent). Many human and animal pathogens are vector-borne and transmitted e.g., by infected hematophagous arthropods, such as mosquitoes or ticks. These include emerging human pathogens such as Crimean–Congo hemorrhagic fever virus (CCHFV) in Turkey [2], West Nile virus (WNV) in Europe and North America [3,4,5,6], Zika virus in South America [7], and *Rickettsia* species (spp.) worldwide [8]. Gathering information about the ecological details of transmission cycles, dispersal mechanisms, and pathogen–vector–host evolution is critical for better understanding of the underlying mechanisms of the emergence of vector-borne zoonotic pathogens. The movement of vectors, migration routes of reservoir hosts, adaptation of vector species to novel regions, and climate change are examples of ecological risk factors for the geographical spread of vector-borne zoonoses. Vector-borne zoonotic infections can only be controlled when understanding the relationship between the pathogen, the vector(s), and the vertebrate host(s).

### 1.3. Bird Biology and Ecology

#### 1.3.1. Migration and Stopover Ecology

Migration is an inherited trait, and the timing of the migration of birds is influenced by internal and external stimuli, such as endogenous rhythms within the bird (e.g., expressed in fat deposition and restlessness) and seasonal changes (e.g., photoperiod, temperature, food supply, and weather conditions). Migration occurs in most bird species that live in seasonal environments, since regular biannual movements are needed in order to find favorable food and weather conditions. Many bird species therefore migrate seasonally along established migration flyways, mainly along north–south routes. The three major flyways are the American flyway, the African–Eurasian flyway, and the East Asian–Australasian flyway, which have been further divided into additional flyways [9]. It should be noted that the major flyways are simplifications. Short-distance migrants can perform their migration route in one or a few single flights. Long-distance migrants, whose migration routes take several months and often include crossing ecological barriers, may break their journeys for hours or up to weeks at a time at stopover sites along the migration route to rest and replenish fuel reserves. Where, when, and for how long they stay at a stopover site will affect their migration success and subsequently the following reproductive success. At some stopover sites, large numbers of individuals gather at the same time. Arrival of many long-distance bird species at breeding and wintering areas usually occurs around the same time each year. The migratory behavior of birds enables natural links between continents and thereby connects different biomes (areas with similar pattern of vegetation, fauna, and climate) and facilitates the transfer of infesting ectoparasites, such as ticks, and avian- and vector-borne pathogens over ecological barriers—such as mountains, deserts, and oceans—and between distant geographical sites [10,11].

#### 1.3.2. Birds as Hosts

Birds may be part of enzootic (constantly present in an animal population in a certain area) transmission cycles and serve as reservoirs (species that maintain pathogen transmission at a low but steady rate) and amplifying hosts (species that increase pathogen transmission) for pathogens. For example, *Borrelia garinii* [12], Sindbis virus [13], WNV [14], and avian influenza viruses [15] are known to have birds as reservoirs or amplifying hosts and to be carried by birds during migration.

### 1.4. Tick Taxonomy, Biology, and Ecology

#### 1.4.1. Tick Taxonomy

Ticks belong to the phylum Arthropoda (which includes spiders, insects, mites, etc.), the class Arachnida, the order Parasitiformes, and the suborder Ixodida [16]. The suborder Ixodida contains obligate hematophagous ectoparasites and is composed of three extant families: Ixodidae (hard ticks), Argasidae (soft ticks), and Nuttalliellidae (one species) [16]. The Ixodidae family includes 742 species [17], while the Argasidae family includes about 190 species [18]. The family Ixodidae is divided into two groups: Prostriata and Metastriata. The Prostriata group contains the genus *Ixodes* and 255 species [17]. The Metastriata group comprises 486 species in 15 genera, e.g., *Amblyomma*, *Dermacentor*, *Haemaphysalis, Hyalomma*, and *Rhipicephalus* [17].

#### 1.4.2. Life Cycle of Ixodidae Ticks

The life cycle of Ixodidae ticks includes the embryonated egg and the three active stages: the larva, the nymph, and the adult. The life cycle is completed when the female tick has laid her eggs in the environment. A new cycle begins with embryogenesis (development and growth of an embryo) of the eggs, followed by the hatching of larvae, which disperse into the vegetation or nest environment to seek hosts or enter diapause (temporary pause in development) for over-wintering. Ticks are hematophagous and require a blood meal from a host to be able to molt into the next stage of life, and the female tick requires a blood meal to lay her eggs. The number of blood meals and hosts required for completion of the life cycle depends on the tick species. Some species require one host, while others require two or three hosts. Most ticks have a three-host life cycle, which means that they seek a host, feed, and drop off to molt into a new stage in the environment. Thus, the active stages feed on separate hosts and are found free-living between feeding periods. In contrast, ticks with a two-host life cycle molt from larva to nymph on the same host. The unfed nymph then re-attaches for a second blood meal on the same host before dropping off. In species of the subgenus *Boophilus* in the genus *Rhipicephalus*, all stages feed on the same host individual. These ticks have a one-host life cycle. Engorgement is completed within several days, depending on tick species and its host [16]. The host-seeking activity of ticks is driven by both abiotic (e.g., temperature and humidity (the amount of water vapor present in the air)) and biotic (e.g., vegetation structure and host abundance) factors. The majority of ticks die from desiccation or starvation.

#### 1.4.3. Feeding Preferences

Most ticks prefer to feed on certain groups of animals, with some being host-specific (specialists) (e.g., *Ixodes trianguliceps* (Birula, 1985)), while others have a wide host range (generalists) (e.g., *Ixodes ricinus* (Linnaeus, 1758), also known as the common tick). Furthermore, different life stages of ticks have different host preferences. Larvae and nymphs often infest smaller mammals and birds, while adults prefer larger mammals [16].

#### 1.4.4. Dispersal Mechanisms

In general, ticks have little mobility, and thus their dispersal (movement to a location where establishment may occur) depends on the movements of hosts, such as wild mammals, livestock, and migratory birds. For example, animal movement and trade likely were involved in the recent introduction of the tick species *Rhipicephalus microplus* (Canestrini, 1888), a vector for tick-borne pathogens (TBPs) of livestock, in West Africa [19]. A wide range of tick species, most commonly of the tick genera *Ixodes* and *Hyalomma* [20], can parasitize wild birds, especially ground-feeding and ground-breeding species [21]. Previous studies have shown that migratory birds can transport ticks carrying human pathogens over long distances [22,23]. Tick species that spend long periods on their host are more likely to be transported over long distances, and migratory birds that pass through a wide range of areas are more likely to encounter a greater range of ticks and tick-borne microorganisms.

#### 1.4.5. Vector Competence

Vector competence is the ability of a vector organism to acquire, maintain, and transmit an infectious agent. Ticks are highly effective as vectors since they:

- inhabit every continent;
- interact with different vertebrate hosts during their life cycle;
- take several blood meals during their life cycle;
- have a relatively long duration of blood feeding;
- remain infectious for long periods of time (including over winter);
- efficiently transmit pathogens both in immature and adult stages;
- have relatively long lifespans (life cycle often measured in years);
- have different modes of transmission: horizontal (passage of pathogen to host during feeding), transstadial (passage of pathogen during the molt to the next life stage), transovarial (passage of pathogen from the female tick to her eggs), and during co-feeding (passage of pathogen from infected tick to non-infected tick while feeding in close proximity on a non-infected host) [24,25].

Besides being vectors for bacteria, viruses, and parasites, ticks can also act as reservoir hosts—a phenomenon made possible by transovarial transmission of pathogens and recorded for *Rickettsia* bacteria [26], tick-borne encephalitis virus (TBEV) [25], and CCHFV [27]. In nature, TBPs are maintained in enzootic cycles involving enzootic tick vectors and wild animals. A bridge (or link) vector can acquire the pathogen from an infective enzootic host and subsequently transmit the pathogen to a secondary host or a human.

#### 1.4.6. Tick-Borne Diseases

Ticks are the most common vectors for human disease after mosquitoes, and ticks transmit a greater variety of pathogenic agents than any other arthropod vector group [28,29,30]. See Table 1 for an overview of a selection of TBPs and tick-borne diseases (TBDs). TBDs are the most widespread and common vector-borne diseases in Europe, and their distribution range has increased since the 1980s [31,32], highlighting the need for increased surveillance of tick populations and TBPs in the region. In Europe, Crimean–Congo hemorrhagic fever (CCHF) [2], Lyme borreliosis (LB) [33], and tick-borne encephalitis (TBE) [34] generate the most concern in humans. In livestock, TBDs such as anaplasmosis, babesiosis, and theileriosis are global problems but most important in tropical and sub-tropical regions, leading to reduction in milk and animal production and resulting in economic losses [35,36].

#### 1.4.7. *Hyalomma marginatum* Species Complex

The genus *Hyalomma*, also known as bont-legged ticks, contains 27 species [63] and includes species of both medical and veterinary importance [36,64]. They are medium- to large-sized ticks with distinct brown and pale bands on the legs [16]. Their eyes and long legs enable them to actively seek their hosts in the adult stage [29]. From a taxonomic perspective, the *Hyalomma marginatum* species complex (*H. marginatum* sensu lato (s.l.)) is one of the most difficult groups. The complex is currently believed to comprise five species: *Hyalomma marginatum* (Koch, 1844), *Hyalomma rufipes* (Koch, 1844), *Hyalomma turanicum* (Pomerantzev, 1946), *Hyalomma glabrum* (Delpy, 1949), and *Hyalomma isaaci* (Sharif, 1928) [65], of which the latter is present on the Indian subcontinent (geographical region comprising Bangladesh, India, Maldives, Nepal, Pakistan, and Sri Lanka). Identification of immature stages to species level is difficult and not advised [66].

#### 1.4.8. *Ixodes ricinus* Species Complex

The *I. ricinus* species complex, also referred to as the *Ixodes persulcatus* complex, comprises closely related *Ixodes* species [67], which have a wide host range and an almost worldwide distribution range, including the Neotropics (South America), Nearctic (North America), Palearctic (Europe and Asia), and the Oriental (Asia) [63,68]. Members of the complex, such as *I. ricinus*, *Ixodes persulcatus* (Schulze, 1930) (also known as the Taiga tick) and *Ixodes scapularis* (Say, 1821) (also known as the deer tick) [68], have a three-host life cycle and are known vectors of several zoonotic pathogens, such as the agents causing TBE, LB, and human granulocytic anaplasmosis (HGA).

### 1.5. Tick-Borne Microorganisms

#### Arboviruses

The vast majority of emerging and re-emerging viral infectious diseases responsible for severe illness are caused by viruses with ribonucleic acid (RNA) genomes. Among these RNA viruses, arthropod-borne (arbo-) viruses, including alpha-, bunya-, and flaviviruses, are important as they can cause fatal disease in domestic animals and humans. The life cycle of arboviruses is complex as it involves both arthropod vectors and vertebrate hosts.

##### Flaviviruses

Flaviviruses (FVs) (*Amarillovirales*; *Flaviviridae*) are enveloped RNA viruses. The genomic RNA is single-stranded (ss), linear, positive-sensed, and encodes a polyprotein. The genome is approximately 11 kilo bases (kb) in length and contains a single long open reading frame (ORF) flanked by 5′- and 3′- terminal non-coding/untranslated regions (NCR/UTRs), which form secondary structures needed for replication and translation. The genome encodes three structural (C, capsid; preM/M, membrane; E, envelope) and seven non-structural (NS) proteins. [69]

FVs have a broad distribution range, including Africa, the Americas, Asia, and Europe [70]. At present, the genus comprises 53 species [71], and most members infect both vertebrate and invertebrate species [72]. The genus is divided into three subgroups: (i) the tick-borne FV group, (ii) the mosquito-borne FV group, and (iii) the no-known-vector FV group [72,73]. The tick-borne FVs (TBFVs) are further divided into three subgroups: (i) the mammalian TBFV (M-TBFV) group, (ii) the seabird TBFV group, and (iii) the probably tick-borne group. The M-TBFV group comprises human and animal pathogens, such as TBEV, Louping ill virus, Omsk hemorrhagic fever virus, Powassan virus, Kyasanur Forest disease virus (KFDV), and Alkhurma hemorrhagic fever virus (AHFV) [73].

##### Orthonairoviruses

The Orthonairovirus genus (*Bunyavirales*; *Nairoviridae*) includes enveloped spherical RNA viruses. The genomic RNA of Orthonairoviruses is ss, negative-sensed, and trisegmented, including the small (S), medium (M), and large (L) segments [74]. The genome is up to 22 kb in length and encodes in the complementary sense form four structural proteins: the nucleocapsid (N) protein encoded by the S segment, the glycoproteins G_N_ and G_C_ encoded by the M segment, and the large polypeptide encoded by the L segment, which encodes the RNA-dependent RNA polymerase [74,75]. The genus includes 41 viruses, mainly tick-borne viruses, such as Nairobi sheep disease virus, Dugbe virus, Hazara virus, and CCHFV [76].

### 1.6. Intracellular Tick-Borne Bacteria

Ticks harbor pathogenic and symbiotic bacteria that require a host cell to replicate, including species of the genera *Anaplasma*, *Coxiella*, *Francisella*, *Midichloria*, *Rickettsia*, and *Spiroplasma* [77,78]. An endosymbiont is a microorganism living inside a host as part of a symbiotic relationship. Tick endosymbionts, mostly from the genera *Coxiella*, *Francisella* and *Rickettsia*, have a close evolutionary relationship with pathogens. Endosymbiotic bacteria present within tick cells may be necessary for host survival by providing nutrients that are missing in the blood meal, such as B-vitamins, and thereby improve the fitness of the tick [79,80,81,82]. Endosymbionts may alter transmission of TBPs, as illustrated by the reduced prevalence of the pathogenic *Rickettsia rickettsii* in the presence of the endosymbiotic *Rickettsia peacockii* in the tick species *Dermacentor andersoni* (Stiles, 1908) [82]. It has been speculated that presence of *Coxiella*-like endosymbionts (CLEs) in the salivary glands of *Amblyomma* ticks could influence maintenance or transmission of the pathogen *Ehrlichia chaffeensis* [83].

## 2. Method

Literature searches were performed primarily in PubMed (https://pubmed.ncbi.nlm.nih.gov; 19 November 2022) and via library sources at Uppsala University, Sweden.

## 3. Conclusions

### 3.1. The African–Palearctic Migration System

The African–Palearctic is a geographical region comprising Africa, Europe, parts of the Arabian Peninsula, and Asia north of the Himalayas. Billions of birds in the African–Palearctic migration system migrate biannually between breeding grounds in the Palearctic (geographical region comprising Africa north of the Sahara, Europe, the temperate parts of the Arabian Peninsula, and Asia north of the Himalayas) and wintering grounds in Africa [84], utilizing the East Atlantic, the Mediterranean/Black Sea, and the Asia/East Africa flyways to pass the Sahara Desert or the Arabian Peninsula (geographical region comprising Saudi Arabia, Yemen, Oman, Kuwait, United Arab Emirates, Qatar, and Bahrain). These three migration routes constitute one of the world’s largest bird migration systems, in which passerines (songbirds belonging to the order Passeriformes) are most common [10,84]. On the African continent, the Sahel region (one of Africa´s eight biomes) is an important transitional zone as well as a wintering area for migratory birds in terms of vegetation and food [85]. It stretches from east to west and is situated between the arid Sahara to the north and the humid savannas to the south. During the northern winter until the departure of Palearctic birds in the spring, areas just south of the Sahara including the Sahel region are very dry due to the seasonal movement of the low-air-pressure belt at the equator [85]. Consequently, many Palearctic birds spend their non-breeding winter season in African regions with low rainfall.

### 3.2. Hyalomma marginatum and Hyalomma rufipes

*H. marginatum* and *H. rufipes* are important vectors and have a two-host life cycle, in which birds, lagomorphs (mammalian order comprising hares, rabbits, and pikas), and insectivores (mammalian order comprising shrews, moles, and hedgehogs) most commonly act as hosts of the immature stages, whereas wild and domestic ungulates (hoofed mammals) act as hosts for the adult stage [65,86]. Larvae molt on the host to become nymphs and remain on the same host up to 22.8–26 days [87,88,89], a period long enough to enable trans-continental (Africa to Europe or Asia) spread by trans-Saharan migrating birds. To date, permanent *Hyalomma* populations have not been recognized in Central and Northern Europe, likely due to the prevailing climatic conditions.

*H. marginatum* (also known as the Mediterranean *Hyalomma*) is known to be present in southern Europe (native in many Mediterranean countries), northern Africa, and parts of Asia [65,86,90,91]. However, the distribution range of the species may expand in the Mediterranean region into Central Europe [92]. Records of the species further south in Africa and north in Europe are likely due to import of immature stages by migratory birds [65,93]. *H. marginatum* infests humans and is the main vector of CCHFV in Europe [60]. *Rickettsia aeschlimannii* [94,95] and *Anaplasma phagocytophilum* [96] have also been associated with the species.

*H. rufipes* (also known as the coarse bont-legged *Hyalomma* or the hairy *Hyalomma*) is a native Afrotropical (zoogeographical region comprising sub-Saharan Africa, Madagascar, and part of the Arabian Peninsula) species that is present in the drier parts of Africa, including sub-Saharan Africa (region south of the Sahara), along the Red Sea coast, and on the Arabian Peninsula [65,86,97]. Records of this species from Europe, many regions of North Africa, and Asia are probably consequences of dispersal of immature stages by migrating birds [65,93,98,99]. The establishment of *H. rufipes* in these regions should be further investigated. *H. rufipes* frequently infest humans and are known to play a role in transmitting pathogens [100,101], being a known vector for CCHFV in Africa [59]. Furthermore, deoxyribonucleic acid (DNA) of *Borrelia burgdorferi* s.l., *Coxiella burnetii, Ehrlichia* species (spp.), and *R. aeschlimannii* has been detected in nymphs collected from birds in Italy [102].

### 3.3. Alkhurma Hemorrhagic Fever Virus

AHFV (also known as Alkhumra hemorrhagic fever virus) was identified in 1995 in Saudi Arabia [103]. Currently, Alkhurma hemorrhagic fever (AHF) is endemic (regularly found in and restricted to a certain region) in several provinces of Saudi Arabia, and the case frequency has increased since 1995, with 620 cases of confirmed AHFV infection reported from 1995 to 2017 [104]. There are also reports from Africa, with four tourist-related cases detected near the Egypt–Sudan border in 2010 and 2013 [105,106,107] and a possible seropositive case as well as cattle-associated *Amblyomma lepidum* ticks testing positive for AHFV RNA detected in Djibouti, situated at the horn of Africa, in 2010 and 2011 [108,109]. The clinical manifestation of AHF resembles that of other viral hemorrhagic fevers, with initial malaise (feeling of discomfort), high fever, headache, arthralgia (joint pain), anorexia, and vomiting followed by encephalitis (inflammation of the brain), jaundice (yellow discoloration of skin, tissues, and body fluids caused by increase in bile pigments in the blood), and ecchymosis (hemorrhagic manifestation, i.e., escape of blood from damaged blood vessels). Rhabdomyolysis (injury of the skeletal muscle resulting in the release of intracellular contents into the circulation) has also been reported [106]. The case fatality rate is reported to be 1 to 25%, with higher rates reported among a low number of hospitalized patients during an early outbreak [110] and later studies extending the disease spectrum by including also asymptomatic infections [111,112]. Currently, there is no specific treatment or vaccine. Surveillance data are limited. The serological status of military staff stationed in different regions of Saudi Arabia was investigated in 2010, and a seroprevalence (AHFV-IgG) of 1.3% was found among the soldiers (*n* = 1024) [113]. Human-to-human transmission has not been reported.

AHFV is closely related to KFDV, a virus endemic in the southwestern part of India and associated with *Haemaphysalis* ticks [114,115]. Ticks, rodents, birds, and monkeys are believed to play a role in the enzootic cycle of KFDV [56,115]. Analysis of full-length genomes has revealed an overall nucleotide sequence diversity of 8.4% between the two viruses [116]. An African origin has been suggested for AHFV and KFDV [117], and it has been suggested that, subsequently, AHFV spread to Saudi Arabia and KFDV to India.

The ecology of AHFV is largely unclear. The virus is believed to be zoonotic, and the tick species *Ornithodoros savignyi* (Audoin, 1827) and *Hyalomma dromedarii* (Koch, 1844) have been proposed as vectors, due to isolation of the virus from the two tick species [55] as well as molecular identification of the complete ORF of AHFV in *O. savignyi* [54], which indicates replication of the virus within the two tick species. Additionally, caprines (goats), ovines (sheep), and camels have been suggested to be involved in the life cycle of AHFV due to their association with the mentioned tick species and their association with AHFV transmission to humans via slaughter and direct contact [54,55,110,118]. The role of birds in the life cycle of AHFV is unknown. The role of northbound AWP birds in the dispersal of AHFV to novel regions has been investigated, and AHFV/KFDV-like RNA has been detected in fully engorged (blood-filled) *H. rufipes* ticks (four nymphs, one larva, and one adult) collected from long-distance migratory bird species with wintering areas in sub-Saharan Africa and breeding areas in Europe and Asia [119]. Since *H. rufipes* is a two-host tick species that molts from larva to nymph on the same host, the engorged larva and nymphs had probably fed only on the bird that they were attached to and could have acquired the virus horizontally from their avian host, transovarially from their mother, or during co-feeding [16,120]. In contrast to the immature stages, the adult tick could have acquired the virus also from a previous host. Due to limited sequence information, it was suggested that the sequences represented AHFV since it is ecologically and geographically more likely, as AHFV is endemic on the Arabian Peninsula, while KFDV is endemic in India [114]. These findings suggest a role for birds in the ecology of AHFV and a potential mechanism for the dispersal of the virus to new regions, including Europe and Asia Minor (the westernmost part of the Asian continent comprising Turkey). Furthermore, these findings together with clinical and serological cases and AHFV-RNA-positive ticks in Africa, suggest a wide distribution range of the virus in Africa and in novel regions, which motivates increased surveillance for this pathogenic emerging zoonotic virus outside of endemic areas.

### 3.4. Crimean–Congo Hemorrhagic Fever Virus

The most important nairovirus with public health impact is CCHFV, which causes a severe and fatal hemorrhagic fever (CCHF) in humans. The clinical manifestation of CCHF initially includes flu-like symptoms (headache, fever, and malaise), arthralgia, diarrhoea, and vomiting [75]. In severe cases, hemorrhage is observed [75]. Most cases of CCHF are asymptomatic or mild, but the case fatality is reported to be up to 30% [60]. Currently, there is no specific treatment or commercially available vaccine.

CCHF is endemic in Africa, southeastern Europe (the known western limit being the Balkans), the Middle East, and Asia [60]. In Europe, CCHF is considered to be a major emerging disease threat. CCHFV strains have been classified into six main genotypes (GTs) based on S segment sequences and the geographical origin: GTs I–III (Africa 1–3 lineages), GT IV (Asia 1–2 lineages), GT V (Europe 1 lineage, Eastern Europe), and GT VI (Europe 2 lineage, Greece), [27,121]—the latter being less pathogenic [122]. In early 2021, the International Committee on Taxonomy of Viruses (ICTV) assigned GT VI into a novel species named Congoid Orthonairovirus, which includes the AP-92 prototype strain [123]. The AP-92 strain was isolated in 1975 from *Rhipicephalus bursa* (Canestrini and Fanzago, 1878) ticks collected in northern Greece and has now been renamed Aigai virus [122].

The ecology of CCHFV includes an enzootic tick–vertebrate–tick cycle. The main vectors and reservoirs of CCHFV in Africa and Europe are the tick species *H. rufipes* and *H. marginatum*, respectively [59,60]. Humans are accidental hosts and acquire the infection via tick bites or by direct contact with viraemic (presence of virus in blood) livestock or patients. The animal reservoir is unknown but the virus has been isolated from both wild and domestic vertebrates, such as hares, bovines (cattle), and canines (dogs) [75]. There is no evidence of disease in domestic animals but anti-CCHFV antibodies have been detected in sera from e.g., bovines, ovines and swine [75], providing evidence of viral exposure. Many birds appear to be resistant to infection with CCHFV [124], but ostriches seem to be more susceptible [125]. Even though many birds do not appear to become viremic, they may play a role in the transportation of ticks carrying the virus. For example, genetic material of CCHFV has previously been detected in *Hyalomma* ticks collected from a migratory bird trapped in Greece [126].

The incidence and geographical range of CCHF have increased during the last decades [127], and autochthonous cases of CCHF have emerged outside of endemic areas in southwestern Europe. In 2016, Spain reported the first human cases [128]. The virus was related to the African 3 lineage of CCHFV (GT III), with a high similarity to the west African Mauritania strain ArD39554 from *H. rufipes* (GenBank accession number: DQ211641) [128], supporting the importation of the virus from northern Africa via infected avian-associated ticks [129]. Detection of genetic material of CCHFV, with probable African origin in adult *Hyalomma lusitanicum* (Koch, 1844) ticks collected in 2010 from red deer (*Cervus elaphus*) in Spain [130], provides further support to an African introduction as well as indication of circulation of the virus in Spain. Since the first Spanish report, 10 additional CCHF cases have been described in Spain from 2013 to 2022 [131,132,133,134].

### 3.5. Anaplasma phagocytophilum

*A. phagocytophilum* (*Rickettsiales*; *Anaplasmataceae*) is a zoonotic intracellular bacterium that replicates within neutrophils and has a wide distribution range, including Europe, the Americas, Africa, and Asia [38,40]. The ecology of *A. phagocytophilum* is complex and involves different vectors and mammalian hosts. Hard ticks of the *I. ricinus* complex are the primary enzootic vectors and bridge vectors of the bacterium to humans: *I. ricinus* in Western Eurasia, *I. persulcatus* in Eastern Eurasia, and *I. scapularis* and *Ixodes pacificus* (Cooley and Kohls, 1943) (also known as the western black-legged tick) in North America [40]. Other tick species may play a role in maintaining enzootic cycles in regions where the main vector is absent [135].

Clinical symptoms are observed in humans and domestic animals, including equines (horses) [136], canines [137], felines (cats) [138], bovines, ovines, and caprines [139]. In domestic ruminants, the disease is called tick-borne fever (TBF) and is characterized by high fever, anorexia, abortion, and drop in milk yield [40], resulting in economic losses for livestock owners in Europe. TBF has not been diagnosed in North America [40]. In equines, canines, and felines, the disease is called granulocytic anaplasmosis (GA) and is characterized by fever and anorexia [40]. The clinical manifestation of HGA ranges from asymptomatic to severe illness with symptoms such as fever, headache, myalgia (muscular pain), and malaise [140]. HGA was first described in the US in 1994 [141], but since then, human cases have been described also in Europe and Asia. In Europe, the reported clinical cases are few, and there are no reports of fatal outcomes, according to the European Centre for Disease Prevention and Control (ECDC) [142]. In contrast, clinical cases are more severe and frequent in North America, with more than 5000 cases reported to the Centers for Disease Control and Prevention (CDC) in 2019 [143] and reports indicating a fatality rate of less than 1% [144]. Underreporting of GA is likely in both humans and animals due to mild or asymptomatic infections. There is no vaccine, but human infections can be treated with antibiotics. Humans are dead-end hosts, i.e., do not have a high enough level of infection to infect a vector, and are therefore not involved in the transmission cycle of *A. phagocytophilum*.

*A. phagocytophilum* has evolved into different strains and genetic variants that display varying pathogenicity and preference of host and/or vector. The genetic diversity of *A. phagocytophilum* has been demonstrated by phylogenetical analyses of several genes, including *groEL* (gene encoding a heat shock protein) [145] and *ankA* [146,147,148]. The *ankA* gene encodes a cytoplasmic protein antigen with ankyrin repeats [149], might be involved in host-specific adaptation [148], and is considered to be a suitable marker for distinguishing variants by host [148,150], allowing potential detection of zoonotic and distinct enzootic cycles.

*A. phagocytophilum* is transmitted transstadially but is not proven to be transmitted transovarially within *Ixodes* species [151], indicating that a vertebrate reservoir host is needed to maintain the enzootic cycle. In North America, the white-footed mouse (*Peromyscus leucopus*) is considered to be the main reservoir host of human variants of *A. phagocytophilum*, while the white-tailed deer (*Odocoileus virginianus*) is considered to be the major reservoir for the non-human variants of *A. phagocytophilum* [37,40]. Knowledge of animal reservoirs of *A. phagocytophilum* in Europe is limited. *A. phagocytophilum* DNA has been detected in wildlife such as roe deer (*Caperolus caperolus*), red deer (*Cervus elaphus*), hedgehog (*Erinaceus europaeus*), and small mammals [152,153,154,155,156], but their reservoir competence (i.e., the ability of an infected host to make an infectious agent available to a vector) remains to be elucidated. *A. phagocytophilum* DNA has been detected in *I. ricinus* ticks and wild or domesticated animals in most European countries [37,38], indicating that the bacterium and the infection are widespread in Europe. Birds have been proposed to have a role as reservoir hosts [157,158], and birds may have a role in the dispersal of ticks infected by the bacterium [158,159,160,161]. The role of northbound spring migratory birds in the dispersal of tick-borne *A. phagocytophilum* in the African–Western Palearctic (AWP) region (AWPR) has been addressed [162]. *A. phagocytophilum* DNA was detected in an *Ixodes* species (sp.) tick found feeding on a trans-Saharan migratory bird trapped at a stopover site in the Mediterranean Sea [162]. The *ankA* sequence of the detected *A. phagocytophilum* variant was found to differ from published sequences, raising questions as to whether it represented a novel variant and whether it could reflect a divergent enzootic cycle of *A. phagocytophilum* with birds as hosts, geographic isolation, or influx from another area by avian migration. Furthermore, the result gives support to the theory of distinct enzootic cycles of *A. phagocytophilum* involving birds and bird-associated ticks [145,163,164]. *A. phagocytophilum* DNA has previously been detected in adult *H. marginatum* ticks collected from animals in France, Israel, and Africa [96,165,166]. However, there is no evidence for immature *H. marginatum* s.l. ticks and birds having a major role in the ecology and northward dispersal of tick-borne *A. phagocytophilum* in the AWPR [162].

### 3.6. Spotted Fever Group Rickettsia

*Rickettsia* are small obligate intracellular bacteria. The genus *Rickettsia* (*Rickettsiales*; *Rickettsiaceae*) is divided into four groups: the ancestral group (comprising *Rickettsia canadensis* and *Rickettsia bellii*), the transitional group (comprising *Rickettsia akari*, *Rickettsia felis*, and *Rickettsia australis*), the typhus group (comprising *Rickettsia prowazekii* and *Rickettsia typhi*), and the spotted fever group (SFG) that includes species such as: *R. aeschlimannii*, *Rickettsia africae*, *Rickettsia conorii*, *Rickettsia heilongjiangensis*, *Rickettsia helvetica*, *Rickettsia honei*, *Rickettsia japonica*, *Rickettsia massiliae*, *Rickettsia monacensis*, *Rickettsia montanensis*, *Rickettsia parkeri*, *R. peacockii*, *Rickettsia philipii*, *Rickettsia raoultii*, *R. rickettsii*, *Rickettsia sibirica*, *Rickettsia slovaca*, and *Rickettsia tamurae* [8,167]. All groups except the ancestral group contain pathogens known to cause human disease. The species *R. aeschlimannii*, *R. conorii*, *R. helvetica*, *R. massiliae*, *R. monacensis*, *R. slovaca*, and *R. sibirica* are considered to be emerging human pathogens in Europe [8,168]. The clinical picture of SFG rickettsioses includes symptoms such as high fever, headache, and rash. An eschar at the inoculation site and neurological manifestation may also develop. The fatality rate of SFG rickettsioses ranges from 0 to 7% [168,169]. Currently, there is no vaccine, but human infections are treated with antibiotics.

SFG *Rickettsia* (SFGR) are transmitted horizontally, transstadially, and transovarially within ticks. Ticks therefore may serve both as vectors and reservoirs for SFGR. SFGR are transmitted to humans mainly by hard ticks of different genera, for example *Hyalomma*, *Ixodes*, and *Rhipicephalus* [8]. The role of vertebrates as reservoir hosts in maintaining zoonotic foci is still unclear. Humans are not involved in the transmission cycle of SFGR and are therefore considered to be accidental or dead-end hosts. SFGR have a worldwide distribution range [8,170], and their range mimics the distribution range of their vectors. Migrating birds have been shown to be bacteremic (presence of bacteria in blood) with SFGR and to carry ticks infected by SFGR [171,172,173], suggesting a role of birds in the ecology and dispersal of SFGR. Northbound migratory birds have been found to be infested by *H. marginatum* s.l. ticks carrying *R. aeschlimannii* [94,174], a SFGR reported to cause infection in humans [175,176].

### 3.7. Francisella tularensis

The genus *Francisella* (*Thiotrichales*; *Francisellaceae*) consists of closely related pathogenic and non-pathogenic species with an intracellular lifestyle. Many of the non-pathogenic species are considered to be endosymbionts [177]. The species *Francisella tularensis* is a facultative intracellular zoonotic bacterium that can multiply within macrophages [178]. It has a broad host range, including mammals, birds, and arthropods [179], and is primarily present in the northern hemisphere. The importance of vertebrate hosts as reservoirs for *F. tularensis* is poorly known. Humans are not involved in the life cycle and are considered dead-end hosts. Transmission to humans has been reported by direct contact with infected animals, arthropods bites (ticks, tabanids (biting flies), and mosquitoes), contaminated water or food, or inhalation of contaminated particles [179]. Three subspecies of *F. tularensis* have been proposed: *F. tularensis tularensis* (Type A), which is endemic in North America, *F. t. holarctica* (Type B), which is found throughout the northern hemisphere, and *F. t. mediasiatica*, which primarily is found in Central Asia [177,180]. Of these, the subspecies *tularensis* and *holarctica* are of clinical importance in humans and responsible for tularemia (also known as rabbit fever) [177]. Tularemia can occur in several forms depending on the route of entry. The most common form of the disease is ulceroglandular tularemia, which presents as a skin ulcer at the site of infection, swelling of regional lymph nodes, aches, and fever [181]. Human infections are treated with antibiotics. *F. tularensis* has a low infectious dose (as few as 10 organisms) [182], can become aerosolized (airborne) and cause a multisystemic disease with a fatality rate of up to 30% [183], and is therefore considered a potential agent of biological warfare [184]. Both subspecies *holarctica* and *tularensis* are highly infectious for humans, stressing the need for both sensitive and specific diagnostic and surveillance methods [177].

Ticks of the genera *Amblyomma*, *Dermacentor*, *Ixodes*, and *Haemaphysalis* are believed to be important vectors [179]. Regarding the genus *Hyalomma*, findings suggest that immature *H. rufipes* do not serve as vectors or contribute to the transmission of *F. tularensis* and that migratory birds do not contribute to the northward dispersal of *F. tularensis*-infected ticks in the AWPR [185]. The genus *Francisella* is currently divided into four major clades (a clade is a group including a common ancestor and all the descendants), and *F. tularensis* is located in Clade 1 [177]. Presence of *Francisella* spp., closely related to *F. tularensis*, in tick species poses a challenge for molecular identification of *F. tularensis*.

### 3.8. Francisella-Like Endosymbionts

*Francisella*-like endosymbionts (FLEs) (*Thiotrichales*; *Francisellaceae*) are intracellular bacteria that are capable of infecting the ovaries of female ticks—a feature that enables transovarial transmission [186,187] and ensures continuation of symbiotic relationships. As the species *F. tularensis*, FLEs are found in Clade 1 of *Francisella* [177]. The genomes of FLEs contain many pseudogenes and inactivated versions of virulence genes of *F. tularensis*, suggesting that FLEs have evolved from a pathogenic ancestor [79,81]. FLEs have a broad distribution range, and they have been found in both hard and soft ticks, including the species *Amblyomma maculatum* (Koch, 1844), *D. andersoni*, *Dermacentor reticulatus* (Fabricius, 1794), *Dermacentor variabilis* (Say, 1821), and *Ornithodoros moubata* (Murray, 1877) [79,188,189,190,191,192,193,194,195,196,197,198]—possibly due to the spread of a pathogenic ancestor among different tick taxa during tick feeding on shared infectious hosts or through co-feeding [197]. In contrast to CLEs, which are considered to be obligate symbionts in most tick species [80,199], FLEs have been suggested to be an alternative obligate symbiont in some tick species [79,200]. An absolute reliance on a symbiont can become detrimental to a tick. The tick can escape the dependency by replacing the old symbiont with a new bacterium obtained from the environment [201]. It has been suggested that FLEs may have replaced CLEs in several tick lineages, including *A. maculatum* and *O. moubata* [79,187,200]. Ticks may depend on FLEs since they provide B-vitamins [79,81]. The knowledge about FLEs is limited since they are difficult to culture and few genomes have been assembled and characterized.

### 3.9. Midichloria

*Candidatus* Midichloriaceae is a novel family within the order *Rickettsiales* comprising intracellular bacteria associated with ticks [202]. The species *Candidatus* Midichloria mitochondrii has an intramitochondrial lifestyle and is considered to be an endosymbiont of the tick species *I. ricinus* [203]. It resides primarily in the ovaries or ovarian primordia (a primordium (singular) is a group of cells that represent the earliest stage in organ or tissue development) of ticks [204], a feature that enables transovarial transmission. The effect *Candidatus* M. mitochondrii has on ticks and its potential to infect mammalian hosts are largely unknown. It has been suggested to be a nutritional endosymbiont because it encodes genes for the production of co-factors and the B-vitamin biotin [205]. *Candidatus* M. mitochondrii DNA has been detected in the salivary glands of ticks [206], DNA of bacteria related to *Candidatus* M. mitochondrii has been detected in different mammalian species, including equines, ovines and canines [207], and anti-*Midichloria* antibodies have been detected in blood samples from humans and canines bitten by ticks [207,208], which could indicate that *Midichloria* bacteria are transmitted horizontally between ticks and vertebrate hosts. Bacteria related to *Candidatus* M. mitochondrii have been detected in other tick species of several tick genera, including *Ixodes*, *Rhipicephalus*, *Amblyomma*, and *Hyalomma* [209,210]. *H. marginatum* s.l. ticks and their trans-Saharan migratory avian hosts trapped in Italy during spring migration have been found to carry *Midichloria* sp. DNA [211]. The presence of *Midichloria* DNA in the avian blood was suggested to be associated with lower fat reserves in the tick-infested birds [211].

### 3.10. Co-Infections/Co-Occurrence of Tick-Associated Microorganisms

Ticks may contain multiple bacterial species and may be co-infected by multiple pathogens [77,212] and thereby potentially transmit several pathogens and cause co-infections in hosts, including humans [213,214]. Multiple infections may show variable clinical symptoms and complicate the diagnosis of TBDs [215]. Co-occurrence of *Francisella* and SFGR in ticks infesting birds migrating from Africa to Europe and Asia during the spring has been investigated, and the findings suggested co-occurrence of *R. aeschlimannii*, FLEs, and *Candidatus* M. mitochondrii in *H. rufipes* [185]. The findings also suggested that migratory birds contribute to the geographical spread of *H. rufipes* containing *Francisella*, SFGR spp., and *Midichloria* in the AWPR [185]. Co-occurrence of FLEs and *Midichloria* may be essential for nutritional symbiosis in *Hyalomma* ticks, including *H. marginatum*, *H. rufipes*, *Hyalomma aegyptium* (Linnaeaus, 1758), *Hyalomma anatolicum* (Koch, 1844), *H. dromedarii*, *Hyalomma excavatum* (Koch, 1844), *Hyalomma impeltatum* (Schulze and Schlottke, 1929), *H. lusitanicum*, and *Hyalomma truncatum* (Koch, 1844) [185,216]. The interaction of FLEs and *Midichloria* with pathogenic bacteria, such as *R. aeschlimannii*, needs to be investigated.

### 3.11. Association between Avian Ecology and Tick Taxon

The ecology of the vertebrate host contributes to the prevalence of tick infestation and the geographical range expansion of ticks. Avian hosts are more likely to be involved in long-distance range expansion of ticks compared to terrestrial hosts due to their migration patterns. Considering that more than a billion birds may cross the Mediterranean Sea during spring migration [84], millions of infesting ticks are likely to be transported from Africa to Europe and Asia each year. Adult *H. rufipes* ticks have been detected outside of their endemic areas, with reports from Central and Northern Europe [93,217,218,219,220]. The presence of adult specimens outside endemic areas is likely a result of avian-associated introduction of immature stages and prevailing weather conditions that allowed progression of the life cycle. Previous findings have suggested that there is an association between bird ecology and tick taxon (biological classification unit), i.e., bird species that share the same habitat as *H. rufipes* during the non-breeding season or spend time in the same habitat as *H. rufipes* during the migration are more likely to become infested by *H. rufipes*, and long-distance migrants are involved in the transportation of immature *H. rufipes* to countries at higher latitudes [221]. With a warmer climate and available hosts for adults, the likelihood of the completion of the life cycle and the establishment of permanent *Hyalomma* populations in the central and northern regions of Europe will increase, highlighting the need for establishing surveillance programs for monitoring the risk of introduction and establishment of *H. rufipes*, including the viral agents CCHFV and AHFV, in the Western Palearctic (WP) (geographic region comprising Europe, North Africa, and parts of the Arabian Peninsula and Asia). Results suggest that migratory bird species wintering in African open habitats and wetlands are good candidates for monitoring the introduction of *H. rufipes* into the WP [221].

### 3.12. Complex Mechanisms Drive the Emergence of Vector-Borne Zoonoses

The mechanisms that drive the emergence of zoonotic infectious diseases are complex, and vector-borne infections are a growing concern globally. The life cycle of vector-borne pathogens involves the pathogen, the vector, and the vertebrate host. Identifying the species that act as vectors, vertebrate hosts, and vehicles of vector-borne zoonotic pathogens is imperative for elucidating the transmission cycles, dispersal mechanisms, and establishment of vector-borne pathogens in nature. The introduction of pathogens and vector species, abundance of vectors and hosts, and adaptation of pathogens to new vectors and vertebrate hosts are determinants in the dispersal and establishment of vector-borne infections. An important geographical determinant of ticks is the climate. With global warming, the distribution range and abundance of ticks could increase in the WP. The influx of millions of African ticks and their microorganisms into the WP via avian transport might therefore pose a greater risk for the public and animal health in the future. Since vector-borne zoonotic infection systems can only be understood by elucidating the details of the systems, future research on tick-borne zoonotic infection systems should have an integrated approach and focus on the systems’ biological, ecological, climatic, clinical, and epidemiological features. Furthermore, the identification of involved vectors, vertebrate hosts, and transmission mechanisms as well as increased awareness and vector control are imperative for the prevention of infectious diseases where there is an underreporting of clinical cases and absence of treatment and/or vaccines, as is the case for AHF, CCHF, and HGA.

## Figures and Tables

**Table 1:** Causative agents, distribution range, vectors, and animal reservoirs of selected tick-borne diseases.

Group	Genus	Species	Disease or Syndrome * (Observed in)	Distribution Range	Vector(s)	Animal Reservoir(s)	References
Bacteria	*Anaplasma*	*A. phagocytophilum*	Human granulocytic anaplasmosis (Human, cattle, goat, sheep, horse, dog, cat)	A, As, E, NA	*Ixodes pacificus* *Ixodes persulcatus* *Ixodes ricinus* *Ixodes scapularis*	R, Ru	[37,38,39,40,41]
	*Borrelia*	*B. afzelii*	Lyme borreliosis (Human, cattle, dog, horse)	As, E	*Ixodes persulcatus* *Ixodes ricinus*	R	[42,43]
		*B. burgdorferi* s.s.	Lyme borreliosis (Human, cattle, dog, horse)	As, E, NA	*Ixodes pacificus* *Ixodes persulcatus* *Ixodes ricinus* *Ixodes scapularis*	R	[39,42,43]
		*B. garinii*	Lyme borreliosis (Human, cattle, dog, horse)	As, E	*Ixodes persulcatus* *Ixodes ricinus*	B, R	[42,43]
		*Borrelia* spp. (e.g., *B. duttonii*, *B. hermsii, B. parkeri*)	Tick-borne relapsing fever (Human)	A, As, E, NA	*Ornithodoros* spp.	H, R	[41,44,45]
		*B. miyamotoi*	Hard tick relapsing fever (Human)	As, E, NA	*Ixodes pacificus* *Ixodes scapularis* *Ixodes ricinus*	B, R	[45]
	*Coxiella*	*C. burnetii*	Q fever (Human, goat, sheep etc.)	A, As, Au, E, NA	Multiple species of different genera	Ru	[39,41,46]
	*Ehrlichia*	*E. chaffeensis*	Human monocytic ehrlichiosis (Human, dog, goat)	NA	*Amblyomma americanum*	Ru	[41,47]
	*Francisella*	*F. tularensis*	Tularemia (Human, hare, rodent, sheep, goat, etc.)	As, E, NA	Multiple species of different genera	U	[39,41,48,49]
	*Neoehrlichia*	*Candidatus* N. mikurensis	Neoehrlichiosis (Human, dog)	As, E	*Ixodes ricinus*	R	[39,50,51]
	*Rickettsia* (SFG)	*R. aeschlimannii*	Unnamed (Human)	A, E	*Hyalomma marginatum* *Hyalomma rufipes*	U	[8]
		*R. africae*	African tick bite fever, LAR * (Human)	A, WI	*Amblyomma hebrum* *Amblyomma variegatum*	U	[8,41]
		*R. conorii*	Mediterranean spotted fever (Human)	A, As, E	*Rhipicephalus sanguineus*	U	[8,39]
		*R. helvetica*	Unnamed (Human)	As, E	*Ixodes ricinus*	U	[8,39]
		*R. massiliae*	Unnamed (Human)	A, Am, As, E	*Rhipicephalus sanguineus*	U	[8]
		*R. parkeri*	Maculatum infection (Human)	Am	*Amblyomma maculatum*	U	[8]
		*R. rickettsii*	Rocky Mountain spotted fever (Human)	Am	*Dermacentor andersoni* *Dermacentor variabilis* *Rhipicephalus sanguineus*	U	[8,41,52]
		*R. siberica*	Siberian tick typhus, LAR * (Human)	A, As, E	*Dermacentor marginatus* *Hyalomma asiaticum*	U	[8]
		*R. slovaca*	Tibola/Debonel * (Human)	As, E	*Dermacentor marginatus*	U	[8]
Parasites	*Babesia*	*B. divergens*	Babesiosis (Human, cattle)	E	*Ixodes ricinus*	R, Ru	[53]
		*B. microti*	Babesiosis (Human)	E, NA	*Ixodes scapularis*	R	[53]
Viruses	Flaviviruses	Alkhurma (Alkhumra) hemorrhagic fever virus	Alkhurma (Alkhumra) hemorrhagic fever (Human)	AP	*Ornithodoros savignyi* *Hyalomma dromedarii*	U	[54,55]
		Kyasanur Forest disease virus	Kyasanur Forest disease (Human, NHP)	IS	*Haemaphysalis spinigera*	U	[29,41,56]
		Louping ill virus	Louping ill (Human, sheep)	WE	*Ixodes ricinus*	Ru	[39,57,58]
		Omsk hemorrhagic fever virus	Omsk hemorrhagic fever (Human)	As	*Dermacentor marginatus* *Dermacentor reticulatus* *Ixodes persulcatus*	U	[39,41,57]
		Tick-borne encephalitis virus	Tick-borne encephalitis (Human, dog)	As, E	*Ixodes persulcatus* *Ixodes ricinus*	R	[39,41,57]
	Ortho- nairovirus	Crimean–Congo hemorrhagic fever virus	Crimean–Congo hemorrhagic fever (Human)	A, As, EE	*Hyalomma marginatum* *Hyalomma rufipes*	U	[39,41,57,59,60]
	Banda- virus	Severe fever with thrombocytopenia syndrome virus /Dabie bandavirus	Severe fever with thrombocytopenia syndrome (Human)	EAs	*Haemaphysalis longicornis*	U	[61,62]

Host: B, birds; H, humans; NHP, non-human primates; R, rodents; Ru, ruminants; U, unknown. Distribution range: A, Africa; Am, the Americas; AP, Arabian Peninsula; As, Asia; Au, Australia; E, Europe; EAs, East Asia; EE, Eastern Europe; IS, Indian subcontinent; NA, North America; WE, Western Europe; WI, West Indies. Abbreviation: LAR, lymphangitis-associated rickettsiosis; SFG, spotted fever group; spp., species; s.s., sensu stricto; Tibola, tick-borne lymphadenopathy; Debonel, Dermacentor-borne necrosis erythema lymphadenopathy; *, syndrome.
